# Incidence Trends of Rheumatoid Arthritis in Korea for 11 Years (2006–2017)

**DOI:** 10.3390/clinpract14060193

**Published:** 2024-11-13

**Authors:** Hanna Lee, Sang-Il Lee, Hyunjin Lim, Hyun-Ok Kim, Rock Bum Kim, Yun-Hong Cheon

**Affiliations:** 1 Division of Rheumatology, Department of Internal Medicine, Gyeongsang National University Hospital, Changwon 51472, Republic of Korea; hanna890117@gmail.com (H.L.); galleonok@hanmail.net (H.-O.K.); 2 Division of Rheumatology, Department of Internal Medicine, Gyeongsang National University Hospital, Jinju 52727, Republic of Korea; goldgu@gnu.ac.kr (S.-I.L.); lhj895@naver.com (H.L.); 3 Regional Cardiocerebrovascular Disease Center, Gyeongsang National University Hospital, Jinju 52727, Republic of Korea

**Keywords:** rheumatoid arthritis, incidence, autoimmune disease

## Abstract

Background/Objectives: Rheumatoid arthritis (RA) is a chronic inflammatory disorder characterized by joint damage. However, no incidence analyses have been conducted on a Korean population since 2013. We aimed to calculate the incidence of RA and examine trends using complete Korean National Health Insurance Service claims data from 2007. Methods: We used 16 years of Korean NHIS claims data from 1 January 2002 to 31 December 2017. Patients were defined as having RA when diagnosed with ICD-10 codes M05 and M06. We set the 5-year period prior to 2006 as disease-free. Results: From 2007 to 2017, the incidence rate of RA was 35 to 43 per 100,000 individuals. The female-to-male ratio was approximately 3–3.5 to 1. The sex-standardized incidence rate was highest in the 60–69 age group but gradually declined, resulting in a reversal in 2017, with the highest incidence rate observed in the 50–59 age group. The incidence of elderly onset RA (EORA) in individuals aged >60 years exhibited a decreasing trend during the study period (age 60–69, −6.45, 95% CI = −8.27 to −4.62, *p* < 0.001; age ≥70, −6.09, 95% CI = −7.66 to −4.53, *p* < 0.001). Conclusions: This study is the first to analyze the incidence trend of RA over an 11-year period. In South Korea, the incidence of RA has shown a decreasing trend since 2011; the same trend was observed in the EORA group. Young-onset RA showed the opposite trend, suggesting that RA is diagnosed earlier, due to the new RA classification criteria.

## 1. Introduction

Rheumatoid arthritis (RA) is a chronic inflammatory disorder that, if left untreated, results in joint deformities, physical disability, and increased mortality in >60% of patients [1]. The life expectancy of patients with RA is shortened by approximately 10 years for men and 11 years for women, compared to healthy controls [2]. However, the continuous development and introduction of new disease-modifying antirheumatic drugs (DMARDs) with high efficacy and few side effects have led to a gradual improvement in mortality rates over the decades. For instance, one study reported a 2.6% annual decrease in incidence-based mortality. Despite these advancements, patients with RA still exhibit a significantly higher mortality rate than the general population and have a poorer prognosis [3,4,5].

Age is a major prognostic indicator of RA [6,7], likely due to the differences in treatment patterns associated with comorbidities and drug efficacy [8,9,10]. Accordingly, RA is now commonly categorized based on the age at diagnosis, with 60 years as the cutoff for young-onset RA (YORA) and elderly onset RA (EORA) [11]. Patients with EORA often face limitations in drug use due to comorbidities, restricting new and conventional DMARDs. Consequently, mortality rates in EORA patients are more than twice as high as those in YORA patients [12]. This finding highlights the importance of early diagnosis and prompt treatment of RA [13].

The onset of RA is attributed to a complex interplay between genetic predisposition and environmental factors, including smoking, periodontitis, and the gut microbiota [14,15]. Genetic factors account for approximately 60% of the risk, leading to variations in prevalence across ethnic groups [16]. Globally, RA affects approximately 0.3–1.0% of the population, with a female-to-male ratio of approximately 3:1 [6,16]. The reported incidence rates range from to 20–70 per 100,000 individuals in North America, 10–20 per 100,000 individuals in Southern Europe, and 40–90 per 100,000 individuals in Japan [17,18,19,20]. A study utilizing Korean National Health Insurance Service (NHIS) claims data reported an incidence rate of 42 per 100,000 individuals [21]. However, this finding is based on 2010 data and may not accurately reflect recent incidence trends [22,23]. We, therefore, aimed to calculate the incidence of RA and examine the trends thereof using Korean NHIS claims data from 2007 to 2017 for incidence updates.

## 2. Materials and Methods

### 2.1. Data Sources and Study Population

This study included data consisting of requests made by medical institutions in Korea to the NHIS to receive fees after providing medical services. Because the NHIS claims dataset contains only de-identified secondary data released for research purposes, this study protocol was approved by the Institutional Review Board of Gyeongsang National University Changwon Hospital (IRB No. 2024-10-018). The data include the serial medical utilization of the entire national population of approximately 50 million people since 2002, and information such as the type of medical institution, date of visit, duration of admission, International Classification of Diseases (ICD)-10 code for diagnosis, type of treatment procedure, and medications. Sociodemographic information, such as sex, age, and province of residence, was also included. Data from 2002 to 2006 were used as the washout period to define newly diagnosed patients with RA.

### 2.2. Definition of RA and Identification of Incident Cases

NHIS claims data do not include clinical features, such as the results of physical examinations, laboratory tests, radiologic images, and symptoms of patients. Therefore, we defined patients newly diagnosed with RA from the NHIS data, regardless of outpatient status and hospitalization, as follows. First, for the period from 2007 to 2017, patients were defined as having RA if diagnosed with ICD-10 code M05 (seropositive RA) and M06 (other RA) in the main diagnosis code or within the four sub-diagnosis codes. Second, among the diagnosed RA patients, those prescribed a biological agent or any DMARDs were included. Only those who had diagnostic codes and were not prescribed RA drugs were excluded from the cohort. The biological agents and DMARDs used are listed in Supplementary Table S1. Third, among the RA patients, those who had not been diagnosed with RA in the previous 5 years or more were defined as incident RA patients in that year. For example, incident RA patients in 2007 were patients without a diagnosis of RA for 5 years from 2002 to 2006, and incident RA patients in 2017 were patients without a diagnosis of RA for 15 years from 2002 to 2016.

### 2.3. Analysis of Incidence

Incident case numbers were extracted based on a previous definition. The crude incidence rate was calculated by dividing the number of patients per year by the midyear population of Korea. We also calculated the proportions by sex and age group (<40, 40–49, 50–59, 60–69, and >70 years).

The age–sex-standardized incidence rate for the total population was calculated to compensate for the difference in the proportion of the population between the age groups and sex by year. At this time, we set the standard population to the midyear population in 2011, and the calculation formula used is as follows:

Age–sex-standardized incidence rate (per 100,000 person-years) for each year = Σ (incidence rate by age groups and sex at each year × number of standard population by age groups and sex) × 100,000/total number of standard populations

To evaluate the linear trend of the adjusted incidence in the total population, sex, and age groups, we performed a linear regression analysis for incidence values by year. SAS version 9.4 (SAS Institute, Cary, NC) and R version 4.01 was utilized for all analyses.

## 3. Results

The annual total incidence rate was the highest in 2011, at 41.9 per 100,000 person-years, followed by 40.8 and 40.0 in 2009 and 2016, respectively (Table 1). The age–sex-standardized incidence rate was the highest in 2009, at 43.1 per 100,000 person-years and was the lowest at 35.4 in 2014. In males, the age-standardized annual incidence rate was highest in 2007, at 20.4 per 100,000 person-years, and lowest in 2008 and 2014, at 16.2 per 100,000 person-years. In females, the age-standardized annual incidence rate was 65.9 per 100,000 person-years, highest in 2009, and lowest in 2017 at 53.6 per 100,000 person-years. According to the analysis of sex-standardized rates by age group, among those under 40 years, the highest was 11.9 per 100,000 person-years in 2016, and the lowest was 6.8 per 100,000 person-years in 2007. For those in their 40s, the highest incidence was in 2015, at 49.0 per 100,000 person-years, and the lowest was in 2007, at 30.5 per 100,000 person-years. For those in their 50s, incidence was highest in 2011, at 85.3 per 100,000 person-years, and lowest in 2008, at 75.3 per 100,000 person-years. For those in their 60s, the highest incidence rate was 143.1 per 100,000 person-years in 2007, and the lowest rate was 76.8 per 100,000 person-years in 2017. For those aged 70 or older, it was highest in 2009 at 110.9 per 100,000 person-years and lowest in 2017 at 54.1 per 100,000 person-years.

**Figure 1:**
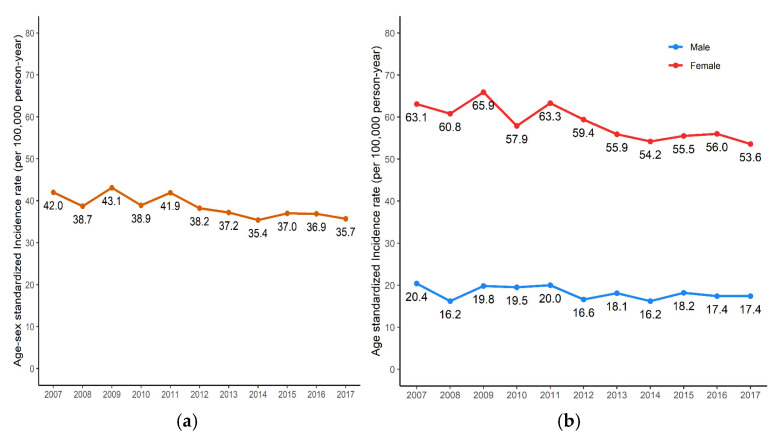
Annual incident rate of RA in Korea: (**a**) The age–sex standardized incidence rate. (**b**) The age-standardized incidence rate.

From 2007 to 2017, the incidence rate tended to decrease to 35.4–43.1 per 100,000 person-years (Figure 1a). The age-standardized annual incidence rate in males was 16.2–20.4 per 100,000 person-years; in females, 53.6–65.9 per 100,000 person-years. Total age–sex-standardized incidence rate showed a similarly decreasing trend. The female/male ratio was 3–3.5, which was similar between 2007 and 2017 (Figure 1b).

The sex-standardized incidence rate for those under 40 years old was 6.8–11.9 per 100,000 person-years, and the sex-standardized incidence rate for those in their 40s was 30.5–49.0 per 100,000 person-years. The sex-standardized incidence rate for people in their 50s was 75.3–85.3 per 100,000 person-years. The sex-standardized incidence rate for people in their 60s was 76.8–143.1 per 100,000 person-years, and, for people over 70 years old, it was 54.1–110.9 per 100,000 person-years. The sex-standardized incidence rate in 2007 was highest in the 60s group but gradually decreased and reversed in 2017, with the incidence rate being highest in the 50s group.

**Figure 2:**
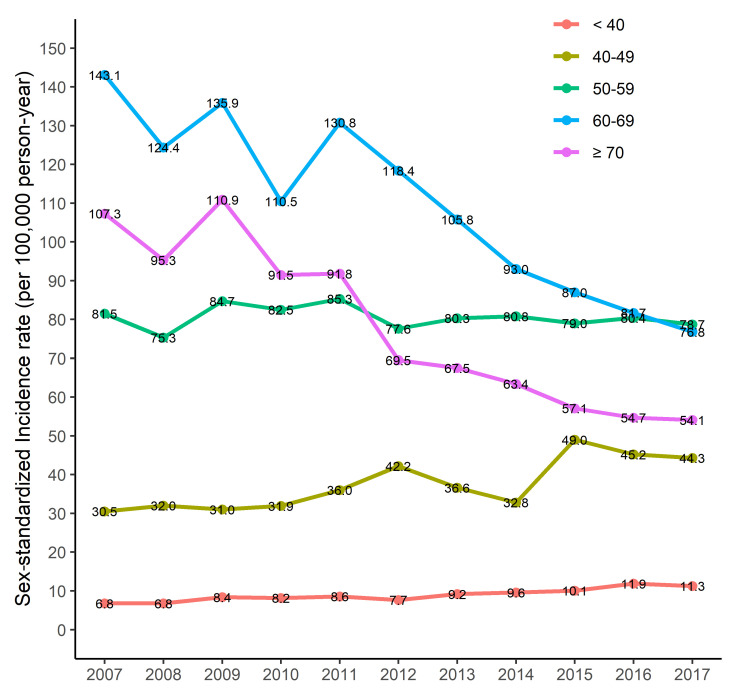
The sex-standardized incident rate of rheumatoid arthritis in Korea.

According to a linear trend analysis of the incidence rate, total incidence was −0.62 (95% CI −0.99 to −0.26) (Table 2). The decreasing trend was greater in women: −0.21 (95% CI −0.53 to 0.10) in males and −1.02 (95% CI −1.55 to −0.49) in females. By age group, incidence tended to increase to 0.47 (95% CI 0.33 to 0.61) in those under 40; 1.62 (95% CI 0.74 to 2.50) in those in their 40s; and −0.17 (95% CI −0.83 to 2.50) in those in their 50s. Incidence showed a tendency to decrease to −6.45 (95% CI −8.27 to −4.62) in those in their 60s and was −6.09 (95% CI −7.66 to −4.53) in those over 70 years of age, with the incidence rate of EORA in those over 60 years of age found to have significantly decreased (Figure 2).

## 4. Discussion

The age-standardized incidence of RA peaked in 2011 at 41.9 per 100,000 person-years, followed by a gradual decline to 35.7 per 100,000 person-years in 2017. Over the past decade, the age-standardized incidence rate ranged from 35.4 and 43.1 per 100,000 person-years. The rates are like those in other Western countries. The incidence of RA in the United States, published using data from 2005 to 2015, was 41 per 100,000 person-years. In Canada, published in 2019, the incidence was 22.7 per 100,000 person-years. In the UK, the incidence rate was 38.1 per 100,000 in 2014 [24,25,26]. Male age-standardized incidence rates exhibited a similar trend, peaking in 2007, and the lowest rates were observed in 2008 and 2014. The female age-standardized incidence rates followed a similar pattern, reaching a maximum in 2009 and a minimum in 2017. The female-to-male incidence ratio remained stable at approximately 3:1 throughout the study period. Our findings align with those of previous studies conducted in Korea, which reported annual incidence rates of 28.5/100,000 and 42.0/100,000, respectively, along with female-to-male ratios of approximately 3:1 [16].

Unlike previous studies, our analysis spans 11 years and provides a more comprehensive understanding of RA incidence trends over an extended period. During the study period, the overall RA incidence showed a declining trend starting from 2011, which was particularly noticeable in the EORA group. This finding implies that, since this study analyzed RA using a code-based definition, the change in diagnostic criteria might have influenced the observed decline in rates. Before 2011, RA was diagnosed using the 1987 ACR criteria. Subsequently, the definition of RA changed with the 2010 ACR/EULAR criteria. Diseases that were considered unclassified inflammatory arthritis under the 1987 ACR criteria were classified as RA from 2011 onwards, as they met the new classification criteria. This new classification is reflected in our results. The overall incidence trends decrease after 2011, and referencing recent studies from Western countries, we hypothesize that lifestyle changes might be associated with this decline. Genetic and environmental factors are involved in the development of RA, with smoking and obesity being representative risk factors. Population-based cohort studies in the UK and USA have shown that smoking and obesity contribute to these trends [24,26]. However, the NHIS claims data used in this study lack lifestyle factors such as smoking and obesity. Therefore, further research is needed to determine whether these factors influenced the declining trends of RA incidence.

Our results differ when compared to Japan, where the onset age of RA is shifting due to the aging population [27]. Korea is predicted to become a super-aged society in 2025, with more than 20% of the population aged 60 years or older [28,29]. The decreasing trend in the incidence of RA is particularly noticeable in EORA, and the incidence is shifting toward YORA. This increasing trend in YORA suggests that RA can be diagnosed earlier. In addition to the 2010 ACR/EULAR criteria classifying RA patients at an earlier stage [30], rheumatoid factors were included in the national health check-up in Korea, making it possible to manage positive rheumatoid factors in advance through education about the disease. Therefore, the gap between symptom onset and diagnosis time may decrease, and the incidence of YORA increase through early diagnosis [22].

Another explanation for the observed decline in EORA incidence is the misdiagnosis of EORA cases as other conditions, particularly polymyalgia rheumatica (PMR). PMR is a common inflammatory disorder in individuals over 50 years of age and is characterized by symmetrical proximal joint (shoulder and hip) pain and stiffness that lasts for several months [31,32]. Due to the frequent involvement of large proximal joints, it can be challenging to distinguish EORA from PMR [33]. Supporting this notion, data from the Health Insurance Review and Assessment Service in Korea showed an increasing trend in newly diagnosed PMR cases between 2008 and 2012, with incidence rates increasing with age [34]. This misclassification could lead to an underestimation of the EORA incidence rates. Further research is needed to investigate the relationship between the misclassification of PMR and RA.

This study has some limitations. Because National Health Insurance (NHI) claims data are only available for patients visiting medical institutions, RA patients who were not diagnosed or untreated in healthcare institutions were not included and may have been underestimated. However, in Korea, the insurance system is mandatory and may cover 90% of the population. Doctors in clinics and hospitals are highly accessible, so representativeness is reliable. The study period is limited to 2017, not reflecting trends from the past 5–6 years. For recent updates, we are planning a new analysis. Also, lifestyle changes may influence the overall decreasing trends, but NHIS claim data does not contain lifestyle factors such as smoking and obesity results. We plan to conduct further research in the future to address this issue. Additionally, in our study, minocycline or cyclosporine were used in the analysis as DMARDs, although they are not currently used. Furthermore, the newly used Tacrolimus is missing from the DMARD list. Therefore, bias may have occurred. However, the DMARDs used in the analysis were for diagnostic exclusion, and Tacrolimus is not a DMARD that can be used immediately at the time of diagnosis according to the Korean medical system, so it would have had a minimal impact on the analysis results.

## 5. Conclusions

The incidence of RA has decreased in South Korea in recent years. Changes in classification criteria have influenced these results. Furthermore, the change in definition has affected the early diagnosis of RA, leading to an increase in diagnoses at younger ages and a decrease in diagnoses at older ages. One potential benefit of early diagnosis is prompt treatment intervention. The development and introduction of novel DMARDs in recent years, along with improved survival rates, could also contribute to a more favorable mortality rate and prognosis for patients with RA compared to the general population.

## Figures and Tables

**Table 1:** Annual incident cases and rate of RA in Korea.

	2007	2008	2009	2010	2011	2012	2013	2014	2015	2016	2017
Incident cases											
Total, No	18,864	18,050	20,744	19,367	21,542	20,202	20,195	19,728	20,967	21,222	20,941
Male, %	23.9	20.7	22.8	24.9	23.6	21.5	24.3	22.9	24.4	23.6	24.3
Female, %	76.1	79.3	77.2	75.1	76.4	78.5	75.7	77.1	75.6	76.4	75.7
Age group, %											
<40	10.2	10.6	11.1	11.3	10.6	9.9	11.6	12.3	11.9	13.7	12.9
40–49	13.7	15.3	13.0	14.4	14.5	18.2	15.9	14.7	20.6	18.6	18.3
50–59	24.7	25.0	25.8	28.6	28.4	28.9	30.9	32.7	30.6	31.1	31.2
60–69	29.2	27.2	26.4	23.4	25.2	24.7	22.7	21.2	19.9	19.7	19.8
≥70	22.3	22.0	23.7	22.2	21.3	18.3	18.9	19.1	17.0	16.9	17.8
Incidence rate (per 100,000 person-years)											
Total, crude	37.6	35.7	40.8	37.9	41.9	39.0	38.8	37.6	39.7	40.0	39.2
Total, age–sex-standardized	42.0	38.7	43.1	38.9	41.9	38.2	37.2	35.4	37.0	36.9	35.7
Male, age-standardized	20.4	16.2	19.8	19.5	20.0	16.6	18.1	16.2	18.2	17.4	17.4
Female, age-standardized	63.1	60.8	65.9	57.9	63.3	59.4	55.9	54.2	55.5	56.0	53.6
Age group, sex-standardized											
<40	6.8	6.8	8.4	8.2	8.6	7.7	9.2	9.6	10.1	11.9	11.3
40–49	30.5	32.0	31.0	31.9	36.0	42.2	36.6	32.8	49.0	45.2	44.3
50–59	81.5	75.3	84.7	82.5	85.3	77.6	80.3	80.8	79.0	80.4	78.7
60–69	143.1	124.4	135.9	110.5	130.8	118.4	105.8	93.0	87.0	81.7	76.8
≥70	107.3	95.3	110.9	91.5	91.8	69.5	67.5	63.4	57.1	54.7	54.1

**Table 2:** Linear trend of adjusted incidence rates during the 11-year study period.

	Estimated Efficient of Changed Incidence During the 11-Year Period	95% CI of Estimated Efficient	*p*-Value
Total	−0.62	−0.99 to −0.26	0.004
Gender			
Male	−0.21	−0.53 to 0.10	0.157
Female	−1.02	−1.55 to −0.49	0.002
Age group			
<40	0.47	0.33 to 0.61	<0.001
40–49	1.62	0.74 to 2.50	0.002
50–59	−0.17	−0.83 to 0.48	0.564
60–69	−6.45	−8.27 to −4.62	<0.001
≥70	−6.09	−7.66 to −4.53	<0.001

## Data Availability

The data presented in this study are available upon request made by medical institutions in Korea to the NHIS.

## Supplementary Materials

The following supporting information can be downloaded at https://www.mdpi.com/article/10.3390/clinpract14060193/s1, Table S1: List and codes of biological agents and disease-modifying anti-rheumatic drugs.
