# Supplementary material for: Incidence Trends of Rheumatoid Arthritis in Korea for 11 Years (2006–2017)
Source: Clin Pract. 2024 Nov 13;14(6):2475–83. doi: 10.3390/clinpract14060193 (PMC11587020; doi:10.3390/clinpract14060193)
Supplement: Supplementary file 1 [file clinpract-14-00193-s001.zip › clinpract-3270182-suppl.pdf]

Supplementary Table S1. Lists and codes of biological agents and disease-modifying anti-rheumatic drugs.

Conventional synthetic names	Codes of NHIS data
**Biologics**	
Etanercept	455801BIJ, 455802BIJ, 455803BIJ, 455830BIJ, 455831BIJ
Adalimumab	488401BIJ
Infliximab	383501BIJ
Golimumab	621232BIJ
Tocilizumab	520430BIJ, 520431BIJ, 520432BIJ, 520433BIJ
Anakinra	517801BIJ
Abatacept	512230BIJ, 512201BIJ
Rituximab	422601BIJ, 422602BIJ
**DMARDs**	
Methotrexate	192101ATB, 192102BIJ, 192103BIJ, 192104BIJ, 192105BIJ, 192107BIJ, 192108BIJ, 192109BIJ, 192110BIJ, 192111BIJ
Hydroxychloroquine	171701ATB, 171702ATB, 171703ATB
Sulfasalazine	232801ATE
Leflunomide	434601ATB, 434602ATB
Azathioprine	112401ATB
Cyclosporin	139201ACS, 139204ACS, 194701ACS, 194702ACS, 139202BIJ
Tofacitinib	627201ATB
Minocycline	195903ACH
Bucillamine	348201ATB
